# Metabolic flexibility and an unusual route for peptidoglycan muramic acid recycling in mycobacteria

**DOI:** 10.1016/j.jbc.2026.113575

**Published:** 2026-09-18

**Authors:** Stefanos Stravoravdis, Bella R. Carnahan, Rebecca A. Gordon, Kimberly Wodzanowski, Karina Havaleshko, Elisabeth Fils-Aime, Rachel Putnik, Stephen Hyland, Catherine Leimkuhler Grimes, M. Sloan Siegrist

**Affiliations:** 1 Department of Microbiology, University of Massachusetts, Amherst, Amherst, Massachusetts, USA; 2 Department of Chemistry and Biochemistry, University of Delaware, Newark, Delaware, USA; 3 Molecular and Cellular Biology Graduate Program, University of Massachusetts, Amherst, Amherst, Massachusetts, USA; 4 Department of Biochemistry and Molecular Biology, University of Massachusetts Amherst, Amherst, Massachusetts, USA; 5 Department of Biological Sciences, University of Delaware, Newark, Delaware, USA

**Keywords:** cell wall, click chemistry, metabolic engineering, metabolic labeling, mycobacteria, N-acetylmuramic acid, peptidoglycan

## Abstract

*De novo* biosynthesis of cell wall peptidoglycan is essential for bacterial viability under many growth conditions and is a well-validated antibiotic target. Although generally not essential for bacterial fitness under standard laboratory growth conditions, peptidoglycan recycling can aid bacterial survival under host or antibiotic stress. Peptidoglycan consists of alternating sugars *N*-acetylmuramic acid (Mur*N*Ac) and *N*-acetylglucosamine (Glc*N*Ac) cross-linked by peptides. Recycling of these sugars can proceed *via* Glc*N*Ac and glucosamine intermediates (*Escherichia coli*-type) or, in the case of Mur*N*Ac, bypass these intermediates altogether (*Pseudomonas*-type). We serendipitously discovered that the pathogen *Mycobacterium tuberculosis* and model organism *Mycobacterium smegmatis* assimilate Mur*N*Ac probes into their peptidoglycan despite lacking the *Pseudomonas*-type machinery that is normally required for incorporation of these molecules. Our data suggest that muramic acids can incorporate into *M. smegmatis* peptidoglycan *via* multiple pathways: an *E. coli*-type route and a non-*E. coli*, non-*Pseudomonas*-type route with Glc*N*Ac but not glucosamine intermediates. These findings reveal metabolic flexibility in mycobacterial cell wall recycling that encompasses a previously undescribed pathway.

Mycobacteria have a complex cell envelope composed of a cell wall peptidoglycan layer connected *via* arabinogalactan to an outer mycomembrane (1). Biosynthesis of these layers is successful drug targets *e.g.,* the first-line antituberculars isoniazid (2, 3) and ethambutol (4) disrupt mycomembrane and/or arabinogalactan formation and the second-line drug d-cycloserine disrupts peptidoglycan formation (5, 6). Cell envelope biosynthesis is generally less vulnerable to inhibition when bacteria adapt to stress by slowing replication (7, 8, 9). Under these conditions, recycling may become more important. In mycobacteria, for example, recycling of the trehalose sugars in the mycomembrane promote fitness in carbon deprivation (10, 11), hypoxia (12), biofilms (13, 14), host cells (10), and *in vivo* (15). In other bacteria, recycling of the peptidoglycan sugars *N*-acetylmuramic acid (Mur*N*Ac) and *N*-acetylglucosamine (Glc*N*Ac) can support fitness during stationary phase (16); protect against some cell wall-acting antibiotics (17, 18, 19, 20); promote biofilm formation (21) and virulence (22, 23, 24); and limit immune activation (25). Studies of when, how, and why mycobacteria recycle their peptidoglycan are in their infancy (26, 27, 28).

Mur*N*Ac is found only in bacterial peptidoglycan. Two major pathways for recycling this sugar have been described; we refer to them here as *Escherichia coli*- and *Pseudomonas*-type for the organisms in which these mechanisms were discovered. In *E. coli*-type recycling, (anhydro)Mur*N*Ac is released from imported muropeptides and/or imported as a free sugar; phosphorylated sequentially or concomitantly, respectively; converted to Glc*N*Ac and glucosamine intermediates, the latter *via* removal of the 2-acetyl by NagA (16, 25, 29, 30, 31, 32, 33, 34, 35, 36, 37) (Fig. 1*A*). Glucosamine intermediates can enter central metabolism or be transformed into UDP-Glc*N*Ac and -Mur*N*Ac for *de novo* peptidoglycan biosynthesis (26, 34, 35, 36, 38, 39, 40, 41, 42). In *Pseudomonas*-type recycling, imported (anhydro)Mur*N*Ac can be transformed more directly into UDP-Mur*N*Ac by AnmK, MupP, AmgK and MurU, bypassing Glc*N*Ac and glucosamine intermediates (17, 18, 19, 43, 44, 45). Some mycobacteria encode homologs for a subset of *E. coli*-type recycling genes, a subset of which have been experimentally verified, but none encode obvious homologs for *Pseudomonas*-type recycling genes (26, 27, 28). Muramic acid isolated from mycobacterial peptidoglycan can bear either *N*-acetyl (Mur*N*Ac; Fig. 1*B*) or *N*-glycolyl (Mur*N*Glyc; Fig. 1*B*) at the 2-position, the proportion of which varies with culture condition (28, 46, 47, 48).

**Figure 1:**
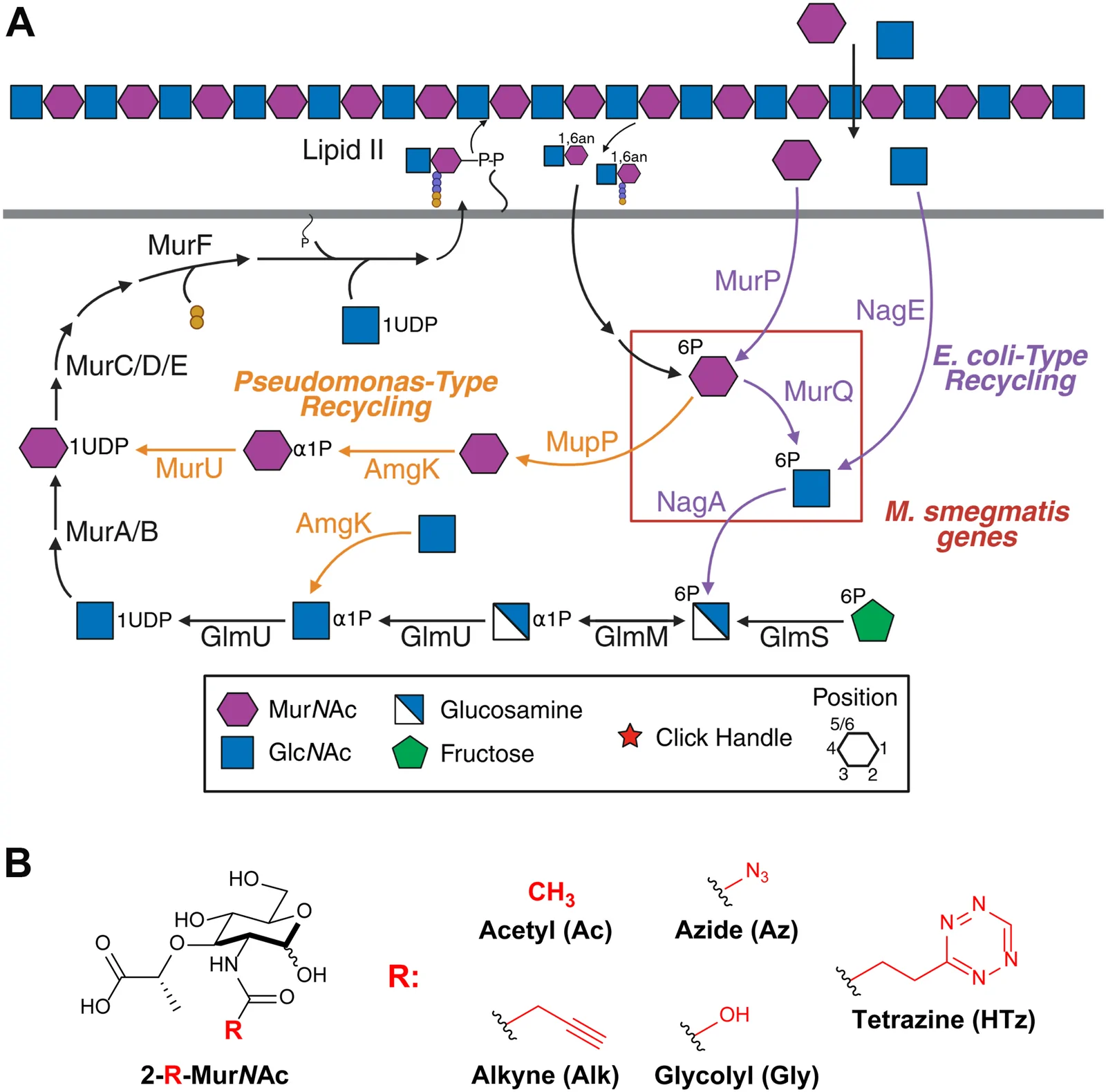
**Known routes for Mur*N*Ac recycling and peptidoglycan biosynthesis.***A,* summary of known mechanisms. In *black* are steps in *de novo* peptidoglycan biosynthesis. Unique steps of *E. coli*- or *Pseudomonas*-type recycling respectively in *purple* and *orang*e. *B,* chemical structures of native and non-native muramic acids used in this work. These structures are known to be processed and recycled *via Pseudomonas*-type recycling (50, 51, 55, 56). MurNAc, N-acetylmuramic acid.

Chemical probes have been useful tools for understanding peptidoglycan metabolism and interactions with the host immune system. Metabolic labeling of the carbohydrate core (49) and the stem peptide (50, 51, 52, 53, 54, 55) enable downstream detection and manipulation. We previously showed that incorporation of Mur*N*Ac probes functionalized at the 2-position requires endogenous or heterologous expression of *Pseudomonas*-type recycling components AmgK and MurU (50, 51, 55, 56). In this study, we unexpectedly discovered that we were able to label members of the Mycobacteriales with 2-modified Mur*N*Ac probes despite the absence of *amgK* or *murU* homologs encoded in their genomes and the presence of an *E. coli*-type recycling enzyme NagA, which normally removes the 2-acetyl. We found that 2-alkyne-Mur*N*Ac (2-alkNAM; Fig. 1*B*) incorporates into both Glc*N*Ac and Mur*N*Ac isolated from the peptidoglycan of the pathogen *Mycobacterium tuberculosis* and the model organism *Mycobacterium smegmatis*. The probe also robustly labels the peptidoglycan of *M. smegmatis* defective for *de novo* production of Glc*N*Ac intermediates. Both Mur*N*Ac and Mur*N*Glyc enhance the growth of *M. smegmatis* defective for *de novo* production of glucosamine intermediates. Taken together, our data suggest that mycobacteria can assimilate muramic acids to bypass multiple steps in peptidoglycan synthesis, including steps that *E. coli*- and *Pseudomonas*-type enzymes are not known to bypass.

## Results

### Corynebacterineae cell surface labeling *via* MurNAc probes

Classic (anhydro) Mur*N*Ac recycling, first defined in *E*. *coli* (35), proceeds *via* (anh)Mur*N*Ac → Mur*N*Ac-6-P → Glc*N*Ac-6-P then loss of the *N*-acetyl at the 2-position, mediated by NagA, to furnish glucosamine-6-P (Glc*N*-6-P; Fig. 1). Glc*N*-6-P can then be funneled into glycolysis or into peptidoglycan biosynthesis, the latter occurring *via* Glc*N*-6-P → Glc*N*-1-P → Glc*N*Ac-1-P → UDP-Glc*N*Ac → UDP-Mur*N*Ac (Fig. 1). An alternative Mur*N*Ac recycling pathway (18, 19, 24, 43, 57), first defined in *Pseudomonas* species, bypasses both Glc*N*Ac and Glc*N* intermediates and proceeds *via* Mur*N*Ac-6-P → Mur*N*Ac → Mur*N*Ac-1-P → UDP-Mur*N*Ac, the latter two steps mediated by AmgK and MurU (Fig. 1). In both the *E. coli*- and *Pseudomonas*-type systems, the nucleotide sugars are processed by additional enzymes to produce the final, lipid-linked precursor lipid II.

We previously synthesized Mur*N*Ac probes functionalized at the 2-acetyl with azide, alkyne, or minimal tetrazine ((50, 51, 52, 53, 55); Fig. 1*B*) and showed that peptidoglycan labeling of bacteria is dependent on the endogenous or heterologous expression of AmgK and MurU. Presumably NagA either does not act on the probes, or deacetylation and potentially other metabolic transformations render the chemical handles undetectable. Surprisingly, we were able to label the model organism *M. smegmatis* using these 2-modified Mur*N*Ac probes (50, 51, 52, 53, 55) followed by detection *via* copper-catalyzed alkyne-azide cycloaddition (CuAAC) or tetrazine ligation to fluorescent labels (Figs. 2 and S1). This was an unexpected phenotype because mycobacterial species lack obvious homologs for AmgK and MurU (27). By contrast, and as expected, *E. coli* labeling occurred only upon heterologous expression of *amgK* and *murU* (Fig. 2). Mur*N*Ac probe labeling of *M. smegmatis* was spatially like that reported for other cell envelope probes (11, 54, 58, 59, 60, 61, 62, 63, 64, 65, 66, 67, 68, 69, 70, 71), including an alkyne derivative of d-alanine-d-alanine (52, 72) (alkDADA; Fig. 2). More specifically, fluorescence derived from 2-alkNAM or alkDADA was enriched at polar and septal sites of elongation in mycobacteria (60, 61, 73, 74, 75, 76, 77, 78). These data suggest that the Mur*N*Ac probes label in the vicinity of active *M. smegmatis* cell envelope metabolism *via* an unknown mechanism.

**Figure 2:**
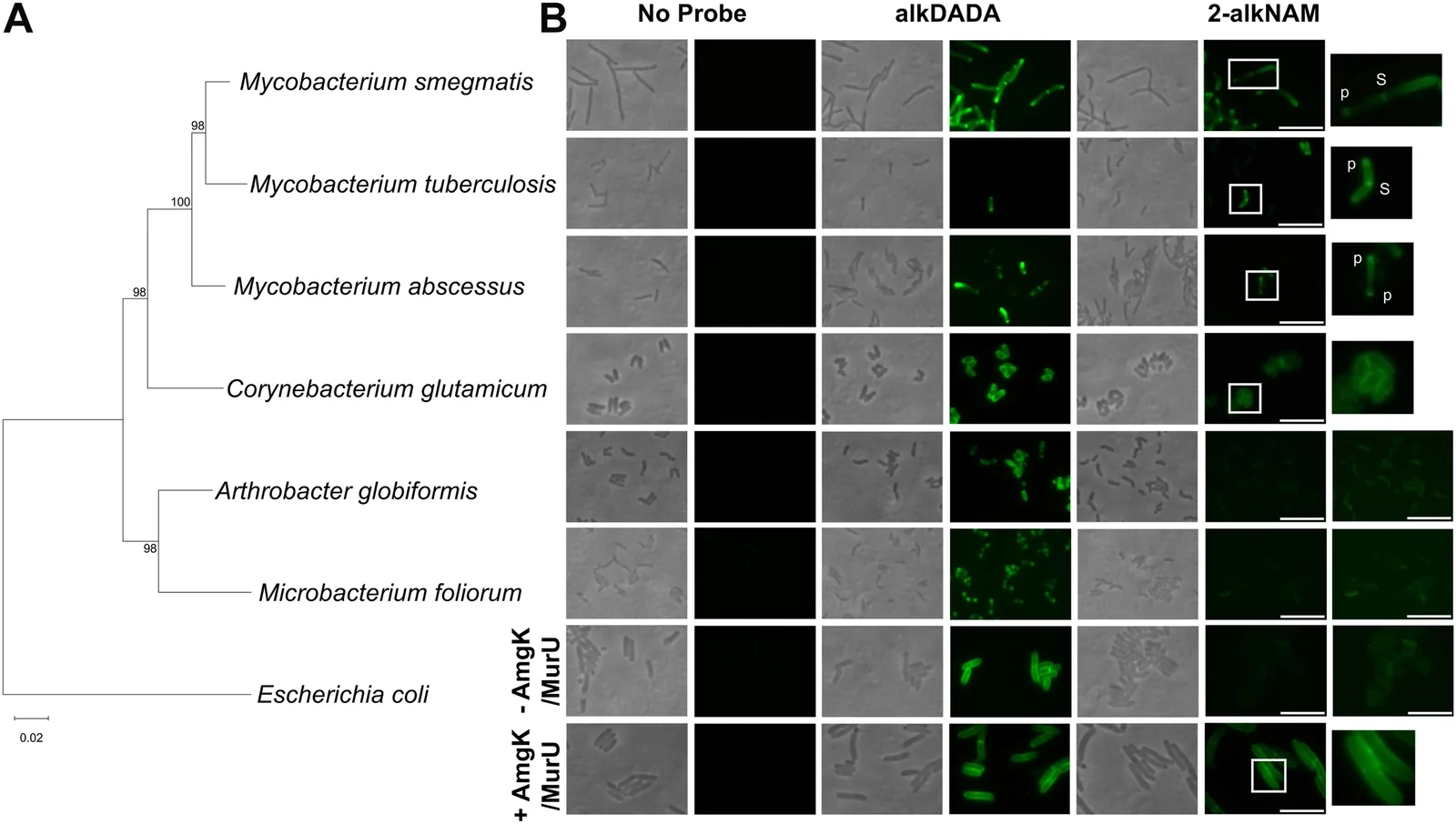
**Cell surface labeling phenotypes across the *Actinomycetes* with cell wall probes alkDADA** (61, 72) **or 2-alkNAM** (50, 51)**.***A,* phylogenetic tree of 16S rRNA sequences. *B,* representative images of bacteria incubated or not with 2 mM alkDADA or 6 mM 2-alkNAM probes (∼25% [*M. smegmatis*] or ∼50% generation time [all other species]) and subjected to copper-catalyzed alkyne-azide cycloaddition with fluorescent azide label. The negative control for 2-alkNAM labeling is *E. coli*, a species that lacks *amgK and murU* homologs; we used the *lptD*4213 K-12 MG1655 mutant, a strain with a known outer membrane defect (79, 101, 102, 103), to enhance permeability of the label (103) and increase sensitivity of detection. The positive control for 2-alkNAM labeling is *E. coli* strain RFM795, another strain with a defect in synthesis of outer membrane proteins (55, 56, 101), which heterologously expresses AmgK and MurU from *Tannerella forsythia*. Zoomed in images of labeled cells or high-exposure FITC images were shown for Mur*N*Ac-treated bacteria (*far right*). Polar and septal labeling is denoted within these zoomed in images as “p” and “s”, respectively. Scale bars, 8 μm. MurNAc, N-acetylmuramic acid.

We next investigated the phylogenetic distribution of Mur*N*Ac probe labeling in species related to *M. smegmatis* that also lack AmgK and MurU homologs. Members of the Mycobacteriaceae family were incubated with alkDADA or 2-alkNAM prior to CuAAC (Fig. 2). *M. tuberculosis* and *Mycobacterium abscessus* labeled with both probes at the poles and septa, like *M*. *smegmatis*, although there remained sizable subpopulations of unlabeled bacteria, particularly for *M*. *abscessus* (Fig. 2). We previously reported cell-to-cell labeling heterogeneity in *M. tuberculosis* for other cell envelope probes that require a fluorophore-appending step for detection (61, 79); we speculate that the phenotype is due to fluorophore permeability rather than underlying cell envelope metabolism. Mur*N*Ac probes also labeled *Corynebacterium glutamicum*, albeit with non-polar localization that was distinct from that of other chemical probes for peptidoglycan (62, 80, 81, 82, 83, 84) (Fig. 2). As *C. glutamicum* is a member of the Corynebacteriaceae family but in the same Mycobacteriales order as the mycobacterial species, we next tested whether the Mur*N*Ac labeling phenotype extended to a different order, the Micrococcales. *Arthrobacter globiformis* and *Microbacterium foliorum* labeled at their poles and septa with alkDADA but had only background fluorescence when treated with 2-alkNAM (Fig. 2). These data suggest that Mur*N*Ac probes can label different members of the Mycobacteriales order and that labeling is spatially coincident with sites of active cell envelope metabolism in the Mycobacteriaceae family.

### MurNAc probe incorporates into mycobacterial peptidoglycan MurNAc and GlcNAc

Mycobacterial species have a homolog of *E. coli*-type NagA but not *Pseudomonas*-type AmgK nor MurU (26, 27, 28). Therefore, these organisms lack a clear mechanism for Mur*N*Ac probe assimilation that would preserve chemical handles installed at the 2-position. However, the polar and septal localization of labeling in *M. smegmatis, M. tuberculosis,* and *M. abscessus* suggested that Mur*N*Ac probes incorporate near or at sites of active cell envelope metabolism. We therefore tested directly whether 2-alkNAM incorporates into mycobacterial peptidoglycan. We treated *M. smegmatis* and *M. tuberculosis* with 2-alkNAM, subjected the bacteria to lysozyme, an enzyme that hydrolyzes β-(1,4) linkages between Mur*N*Ac and Glc*N*Ac, and analyzed by LC-MS. We found that the alkyne was present in lysosome-digested peptidoglycan fragments for both organisms after incubation with 2-alkNAM and not unmodified Mur*N*Ac control (Figs. 3, *A*–*C*, S2–S10). Unexpectedly, some of the digested fragments contained both alkyne and *N*-glycolyl modifications, suggesting that the Glc*N*Ac was modified with the alkyne while the Mur*N*Ac retained the canonical *N*-glycolyl modification (Figs. 3, *A*, *Bi*, *Bii*, S2, S8 and S9). Thus, *M. smegmatis* and *M. tuberculosis* can assimilate 2-alkNAM into both Mur*N*Ac and Glc*N*Ac of their peptidoglycan.

### *E. coli-type recycling can assimilate MurNAc* and *MurNGlyc* in *M. smegmatis*

The presence of both alkyne-labeled Mur*N*Ac and Glc*N*Ac in the peptidoglycan of 2-alkNAM-labeled *M*. *smegmatis* and *M. tuberculosis* (Fig. 3) implied that the probe can incorporate into mycobacterial peptidoglycan *via* Glc*N*Ac intermediates, as UDP-Glc*N*Ac is converted into UDP-Mur*N*Ac and both are then used to construct lipid II. However, this was a puzzling inference as mycobacteria encode *E. coli*-type NagA, which removes the 2-acetyl of Mur*N*Ac to generate Glc*N*-6-P en route to UDP-Glc*N*Ac, and *M. smegmatis* additionally encodes *E. coli*-type MurQ, which converts Mur*N*Ac-6-P to Glc*N*Ac-6-P to generate the substrate for NagA. We therefore tested whether muramic acid recycling in *M. smegmatis*, as in *E. coli*, can proceed *via* glucosamine intermediates. We reasoned that *E. coli*-type recycling may assimilate Mur*N*Ac poorly in the absence of *murQ* and/or when the *N*-acetyl is modified with an alkyne or swapped for an *N*-glycolyl. Because *E*. *coli*-type recycling of exogenous Mur*N*Ac generates Glc*N*-6-P, it can bypass the requirement for *de novo* synthesis of this metabolite by the essential enzyme GlmS (Fig. 1*A*, (16, 26, 34, 35, 36, 40, 41, 42)). We therefore used CRISPR interference (CRISPRi) to knock down *glmS* in WT or Δ*murQ M. smegmatis* and asked whether we could rescue bacterial growth by addition of different muramic acids to the growth medium. In this system, the expression of both d*cas9* and the guide RNA is under the control of an anhydrotetracycline (ATC)-inducible promoter (85, 86, 87). Growth inhibition of WT mycobacteria in the presence of Mur*N*Ac, an acidic sugar, was alleviated by MOPS buffering (compare Fig. S11 to Fig. 4, *Ai*, *Aii*, *Di*, *Dii*, see also Experimental procedures) so we performed subsequent, long-term growth experiments using MOPS-supplemented 7H9 medium. Depletion of peptidoglycan biosynthetic enzymes resulted in low turbidity readings with high variability, the latter of which we attribute to debris from lysed cells. Consistent with a functional *E. coli*-type recycling pathway, we found that exogenous Mur*N*Ac rescues growth of *M. smegmatis* carrying a *glmS*-targeting guide in the presence of ATC (Fig. 4*Aiii*). Rescue was lost in the absence of *murQ* (Fig. 4*Aiv*) or when the 2-position of Mur*N*Ac was modified with an alkyne (Fig. 4*B*). Contrary to our expectations, however, rescue was apparent when the 2-acetyl was swapped for a glycolyl (Fig. 4*C*). These data suggest that *E. coli*-type recycling is operative in *M. smegmatis* and can assimilate Mur*N*Ac or Mur*N*Glyc. The inability of 2-alkNAM to rescue GlmS-depleted *M. smegmatis* may indicate that MurQ and/or NagA is unable to process the probe. Alternatively, or additionally, 2-alkNAM may be toxic in the absence of the GlmS, regardless of whether it is incorporated into peptidoglycan.

**Figure 3:**
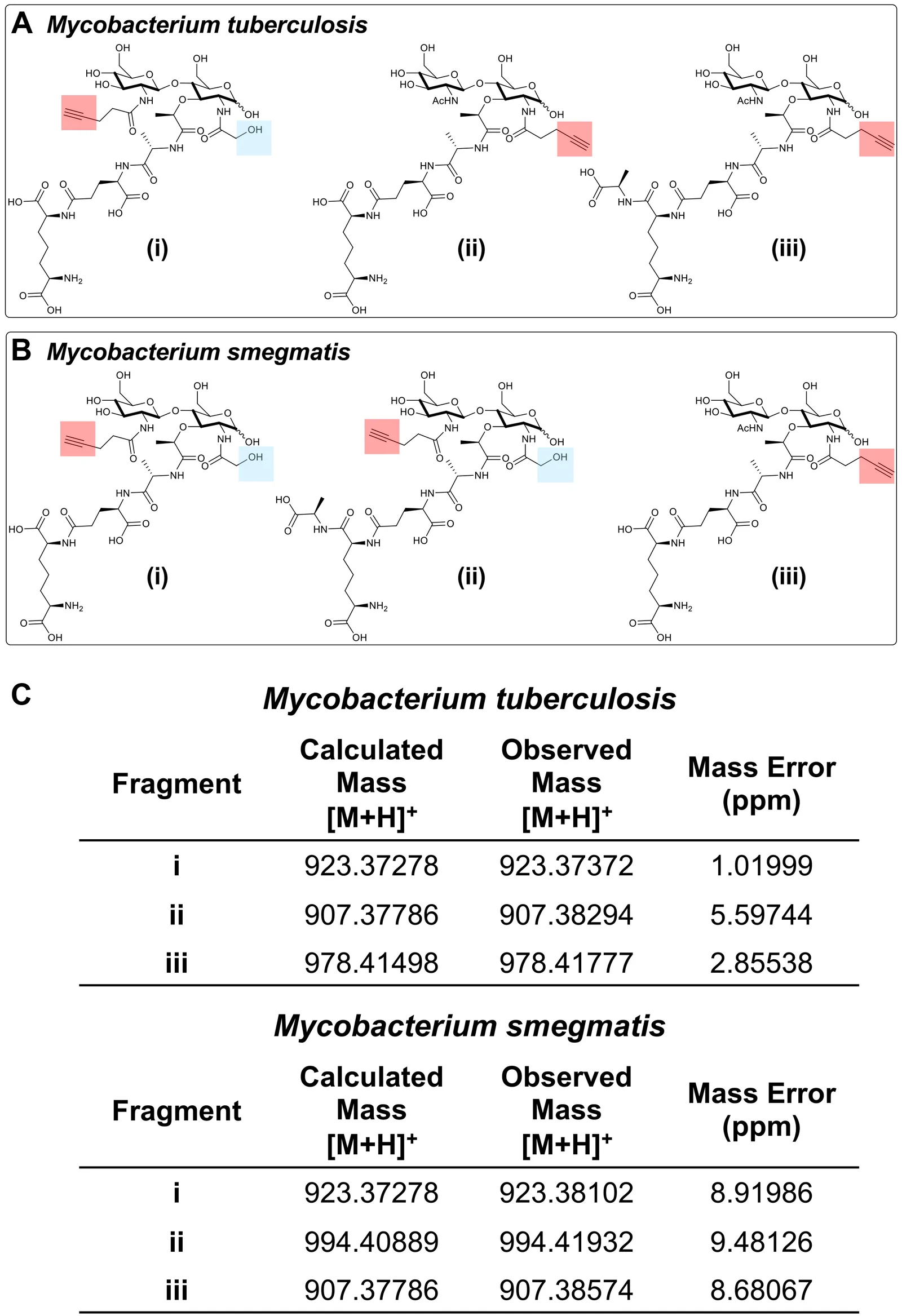
**Proposedchemical structures of disaccharide fragments observed byhigh-resolutionmass spectrometry.** Fragments were produced by lysozyme digestion of 2-alkNAM-treated *M. tuberculosis (A)* and *M. smegmatis* sacculi *(B)* grown in 7H9 medium. *C*, Tabulated data from high-resolution mass spectrometry of observed fragments from *(A* and *B)*. Presence of the alkyne handle (shown in *red)* was detected in *N*-glycolyl-modified disaccharide (*N*-glycolyl shown in *blue*) in Glc*N*Ac (*3Ai*, *3Bi*-*3Bii*) as well as in Mur*N*Ac (*3Aii*-*Aiii*, *3Biii*) for both species. Complete spectra in Supporting Information. GlcNAc, N-acetylglucosamine; MurNAc, N-acetylmuramic acid.

**Figure 4:**
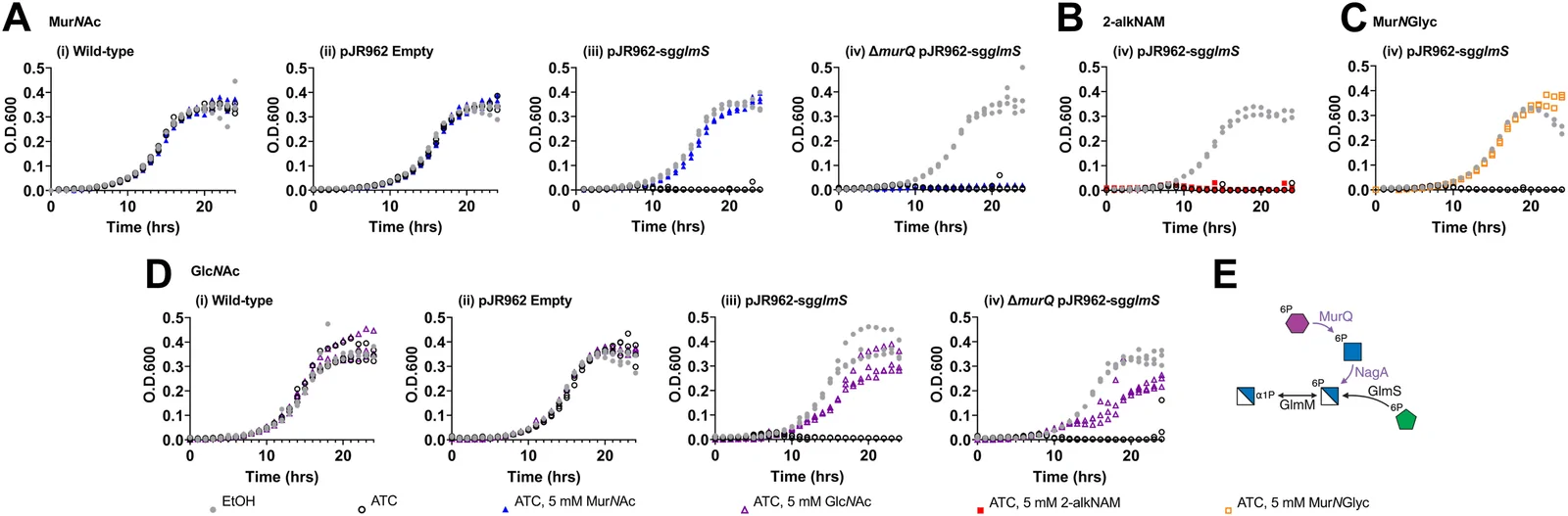
***E. coli*-type recycling pathway can assimilate Mur*N*Ac, Mur*N*Glyc, and Glc*N*Ac in *M. smegmatis.*** WT or Δ*murQ M. smegmatis* ± pJR962 plasmid *(empty)* or containing *glmS* guide (sg*glmS*) and d*cas9* under an ATC-inducible promoter were treated ± 50 ng/ml ATC or ethanol (EtOH) carrier control and supplemented with the indicated amounts of Mur*N*Ac *(A)* or Glc*N*Ac *(D)* at 0 and 4 h of growth in MOPS-buffered 7H9 medium. Representative data shown from two biological replicates performed in technical duplicate or triplicate. Each individual technical replicate is plotted to show the change in mycobacterial growth in response to the shown treatments. *M. smegmatis* pJR962-sg*glmS* was additionally treated ± 50 ng/ml ATC or EtOH and supplemented with muramic acids *(B* and *C)* as described above. Representative data without error bars shown from two biological replicates performed in technical duplicate. *E,* summary of *E. coli*-type muramic acid recycling, as found in *M. smegmatis*. *ATC, anhydrotetracycline;* GlcNAc, N-acetylglucosamine; MurNAc, N-acetylmuramic acid.

### *E. coli*-type recycling assimilates GlcNAc in *M. smegmatis*

*M*. *smegmatis*, in contrast to other mycobacterial species, can recycle Glc*N*Ac and encodes the proteins to do so *via* an *E. coli*-type pathway that does not require MurQ: Glc*N*Ac → Glc*N*Ac-6-P (NagE) → Glc*N*-6-P (NagA; Fig. 1 (26, 27, 28)). Consistent with an operative pathway for *E. coli*-type Glc*N*Ac recycling, we found that exogenous Glc*N*Ac could rescue growth of *glmS*-depleted *M. smegmatis* but, in contrast to Mur*N*Ac, did so in a MurQ-independent fashion (Fig. 4, *Diii* and *Div*).

### Non-*E. coli*, non-Pseudomonas-type recycling pathway can assimilate muramic acids in *M. smegmatis*

While our growth rescue experiments suggested that *M. smegmatis* could transform Mur*N*Ac and Mur*N*Glyc *via E. coli*-type recycling into Glc*N*-6-P (Fig. 4*C*), our analysis of peptidoglycan from 2-alkNAM-treated *M. smegmatis* and *M. tuberculosis* was consistent with a pathway that uses Glc*N*Ac but not glucosamine intermediates (Fig. 3). We therefore hypothesized that muramic acids could also access the *de novo* pathway for peptidoglycan synthesis at one or more steps after GlmS production of Glc*N*-6-P but prior to MurA production of UDP-Mur*N*Ac (Fig. 1). Glc*N*-6-P is converted to Glc*N*-1-P by GlmM then to Glc*N*Ac-1-P and finally UDP-Glc*N*Ac by the bifunctional enzyme GlmU (88, 89)). Bypass of GlmM or GlmU depletion by exogenous muramic acids would respectively support models for recycling of the sugar into Glc*N*Ac-1-P or UDP-Glc*N*Ac. Using CRISPRi to knock down GlmM, GlmU, or MurA in *M. smegmatis* (Fig. S12, *A* and *B*)*,* we found that exogenous Mur*N*Ac or Mur*N*Glyc rescues ATC-mediated depletion of GlmM but not GlmU, nor, as expected, MurA (Fig. 5, *A*–*C*). Together with our biochemical data (Fig. 3), these data suggest that *N*-acetyl and *N*-glycolyl muramic acids can be recycled into mycobacterial peptidoglycan *via* Glc*N*Ac intermediates.

**Figure 5:**
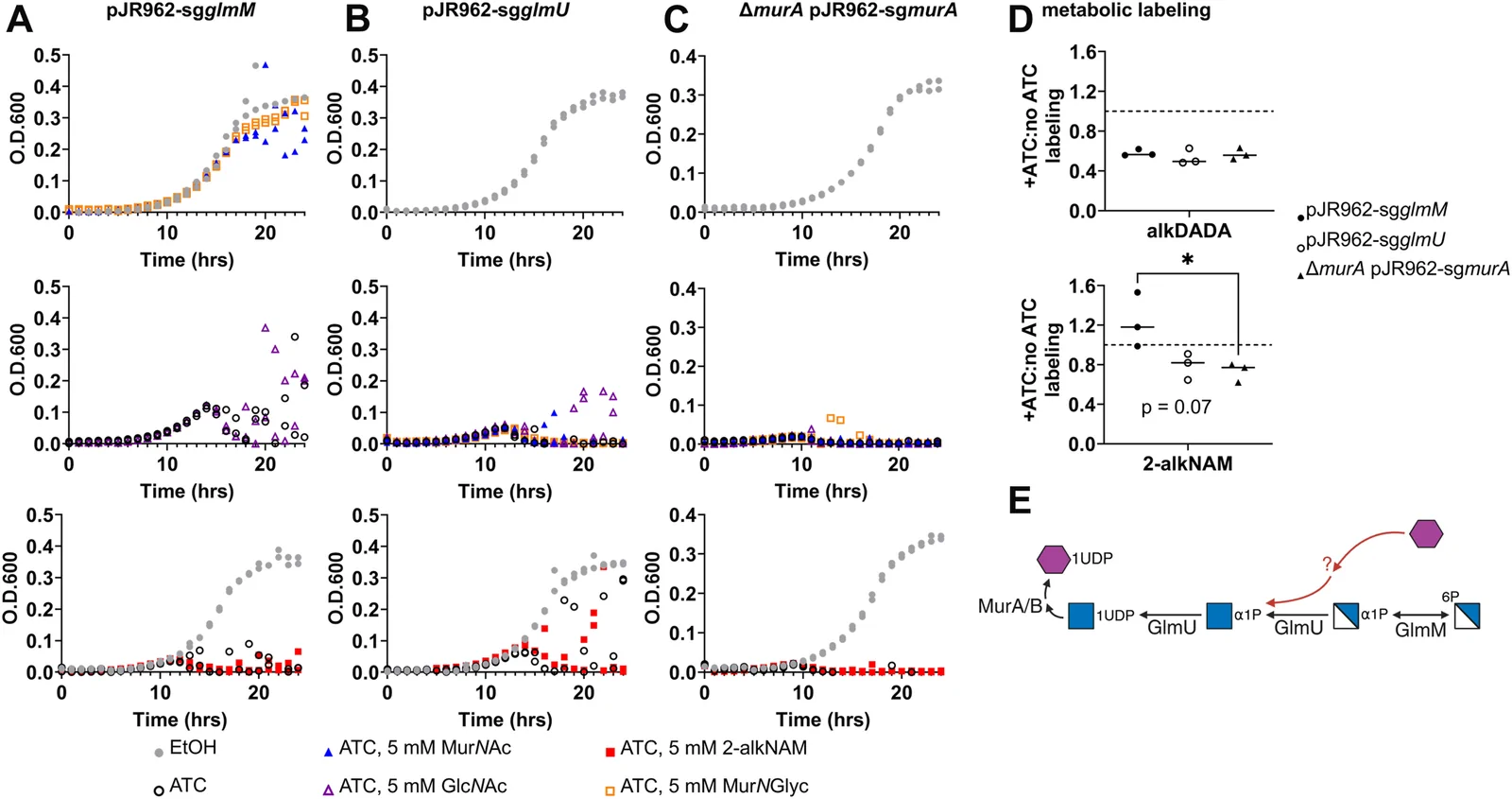
**Muramic acids can be assimilated into *M. smegmatis* peptidoglycan by non-*E. coli*, non-*Pseudomonas*-type recycling pathway.** WT or Δ*murA M. smegmatis* ± pJR962 plasmid containing d*cas9* and guides for *glmM* (sg*glmM*; *(A)*), *glmU* (sg*glmU*; *(B)*)*,* or *murA* (sg*murA*; *(C)*) under an ATC-inducible promoter were treated ± 50 ng/ml ATC or ethanol (EtOH) carrier control and supplemented with 5 mM of Mur*N*Ac, Mur*N*Glyc, Glc*N*Ac, or 2-alkNAM at 0 and 4 h of growth. Representative data shown from two biological replicates performed in technical duplicate. Each individual technical replicate is plotted to show the change in mycobacterial growth in response to the shown treatments. *D,* metabolic labeling with alkDADA *(top)* or 2-alkNAM *(bottom)* probes at 1 h after first evidence of drop in bacterial turbidity. Presence of probes was detected by copper-catalyzed alkyne-azide cycloaddition ligation to azide-fluorophore after fixation. 30,000 events were recorded per sample and ratios of median fluorescence intensities ± ATC were plotted, with each dot representing a biological replicate performed in technical duplicate. Significant differences in labeling *via* one-way ANOVA (*p* < 0.05) are indicated by ∗. *E,* summary of non-*E. coli*, non-*Pseudomonas* mechanism of muramic acid recycling. *ATC, anhydrotetracycline;* GlcNAc, N-acetylglucosamine; MurNAc, N-acetylmuramic acid.

Exogenous Glc*N*Ac failed to rescue GlmM-depleted *M. smegmatis* (Fig. 5, *A*–*C*), suggesting that bypass could be specific to muramic acids. However, in contrast to Mur*N*Ac and Mur*N*Glyc, 2-alkNAM did not rescue the growth of GlmS- or GlmM-depleted *M. smegmatis* (Figs. 4*B* and 5*A*). This was surprising given that WT mycobacteria label with 2-alkNAM (Fig. 2) and assimilate the probe into both Mur*N*Ac and Glc*N*Ac of their peptidoglycan (Fig. 3). Like Mur*N*Ac and Mur*N*Glyc, 2-alkNAM also did not rescue the growth of GlmU- or MurA-depleted *M. smegmatis* (Fig. 5, *B* and *C*). We reasoned that the failure of 2-alkNAM to rescue the growth of any of the depletion strains may be explained by metabolic toxicity rather than by a divergent incorporation mechanism from that of the native sugars. More specifically, we expected that WT mycobacteria incubated for several generations in 2-alkNAM would have lower proportion of alkyne-labeled peptidoglycan precursors compared to strains depleted for *de novo* biosynthetic enzymes due to different pool sizes of endogenous Mur*N*Ac and Glc*N*Ac metabolites. While long-term 2-alkNAM incubation (∼20 h) might initially support peptidoglycan biosynthesis in the absence of one or more biosynthetic enzymes, the resulting cell wall would consist primarily of alkyne-modified sugars and may not be compatible with long-term viability. Therefore, as an orthogonal test of whether 2-alkNAM could bypass GlmM, GlmU, or MurA depletion, we monitored peptidoglycan labeling following short-term probe incubation (30 min). We and others have shown that dipeptide d-amino acid probes incorporate into peptidoglycan and/or lipid II. Specifically, incorporation of dipeptide probes occurs *via* the last cytoplasmic step of the lipid II biosynthetic pathway, MurF (61, 72, 90, 91). Depletion of GlmU or MurA decreased both alkDADA and 2-alkNAM labeling (Fig. 5*D*), as expected from a general slowdown in peptidoglycan biosynthesis and failure of the probes to bypass the loss of either enzyme. By contrast, depletion of GlmM decreased alkDADA but not 2-alkNAM labeling (Fig. 5*D*), respectively consistent with dampened peptidoglycan biosynthesis and GlmM depletion bypass (Fig. 5*E*). The persistence of 2-alkNAM labeling and Mur*N*Ac- and Mur*N*Glyc-enhanced growth of GlmM-depleted *M. smegmatis* are consistent with the notion that muramic acids can be recycled directly into Glc*N*Ac intermediates, most likely entering *de novo* synthesis as Glc*N*Ac-1-P.

## Discussion

Previous *in silico* analysis and experimental characterization (26, 27, 28) revealed that mycobacteria encode a limited subset of *E. coli*-type recycling proteins for peptidoglycan sugars, *e.g.*, NagA, NagC, NagZ/LpqI, and, in the case of *M. smegmatis*, MurQ. Beyond these studies, the mechanisms by which mycobacteria recycle Mur*N*Ac are largely unclear. Given the presence of NagA, which deacetylates the 2-position of Mur*N*Ac, and the apparent absence of a *Pseudomonas*-type recycling pathway to bypass MurA/MurB (lack of AmgK or MurU homologs), we were initially surprised that various mycobacterial species incorporate 2-modified Mur*N*Ac probes into their peptidoglycan (Figs. 2, 3 and S1). The presence of the alkyne handle from 2-alkNAM in both Mur*N*Ac and Glc*N*Ac of WT mycobacteria (Figs. 3, S2–S7), the ability of native muramic acids to support *M. smegmatis* growth in the absence of GlmM (Fig. 5, *A*–*C*), and the persistence of 2-alkNAM labeling in the same background (Fig. 5*D*) all point to a model in which recycled muramic acids can enter *de novo* peptidoglycan biosynthesis as Glc*N*Ac-1-P (Figs. 5*E* and 6).

**Figure 6:**
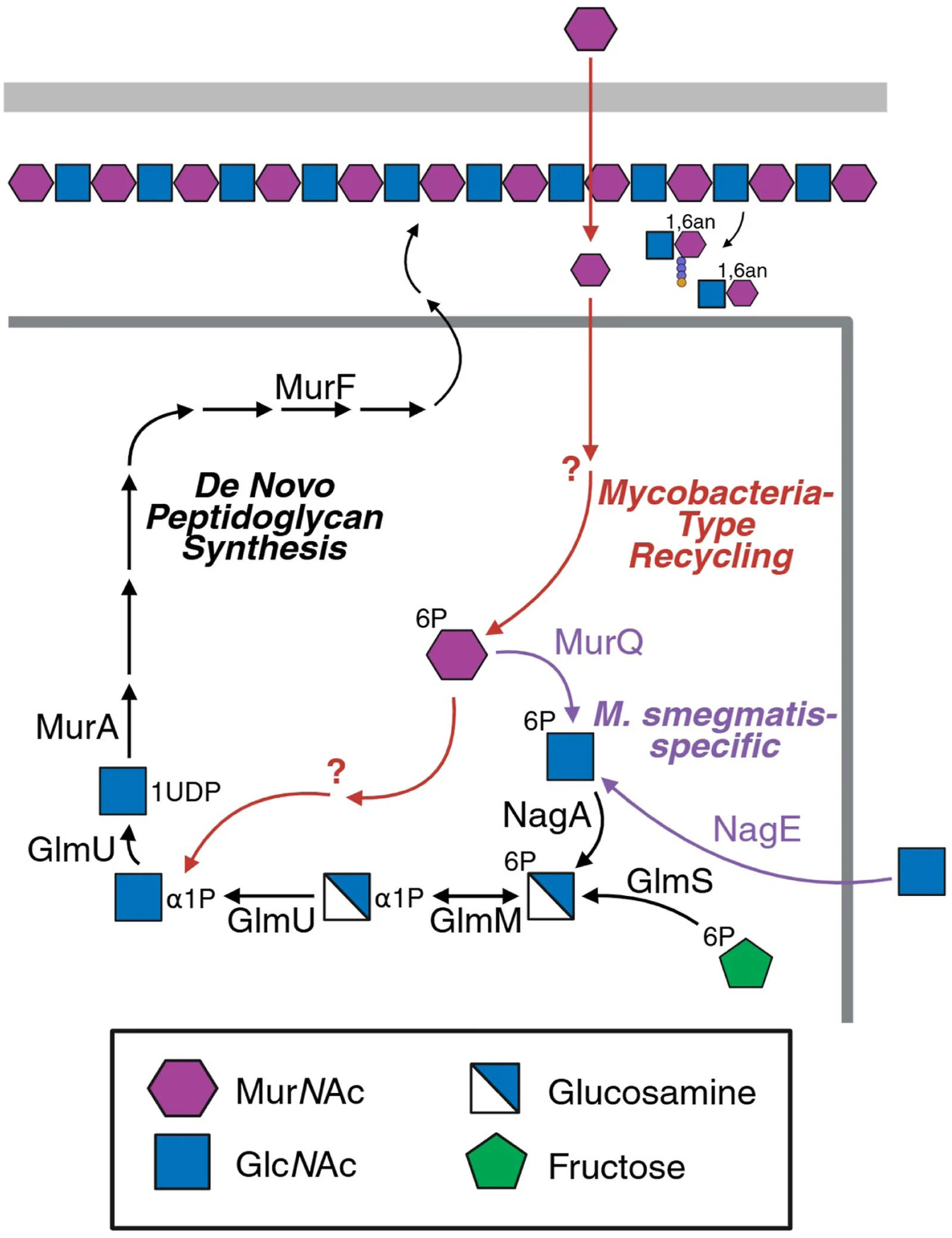
**Multiple pathways for muramic acid recycling in *M. smegmatis***.

*M*. *smegmatis* has additional *E. coli*-type recycling proteins that are not present in other members of the Mycobacteriales, including NagE, AnmK, MurK, MurQ, and NagB (27, 43). However, *M. tuberculosis* and *M. abscessus* also label with Mur*N*Ac probes and do so at the known polar and septal sites of active cell envelope metabolism (Fig. 2). For *M. tuberculosis* we have confirmed that the alkyne handle from 2-alkNAM is present in peptidoglycan fragments. Thus, we favor a model in which Mur*N*Ac probes are incorporated into peptidoglycan *via* a similar mechanism across mycobacteria.

*C. glutamicum* also labels with Mur*N*Ac probes, but in contrast to other cell envelope probes, including d-amino acid dipeptides (61, 72, 90), the fluorescence is evenly distributed around the cell periphery (Fig. 2). We previously reported that peptidoglycan probes localize in part to the sidewall in mycobacterial species, in addition to the poles and septa, and that sidewall labeling is enhanced upon cell wall damage (61, 63, 67). We speculate that the early steps of Mur*N*Ac recycling in *C. glutamicum* are like those of mycobacterial species but that the later steps are divergent, such that Mur*N*Ac probe labeling in *C. glutamicum* reports cell wall repair instead of, or in addition to, cell wall expansion. It is also possible that labeling is non-specific in this organism. Given that we have not yet tested these hypotheses, we refer to the Mur*N*Ac recycling pathway(s) described herein as “Mycobacteria-type” instead of “Mycobacteriales-type.”

Mur*N*Ac can be used as a sole carbon source in different mycobacterial species, including *M. tuberculosis* and *M. smegmatis* (27). Mur*N*Ac processing likely occurs *via* a mechanism in which an unidentified lactyl etherase acts on the sugar to produce Glc*N*Ac and lactate, the latter entering central metabolism. The fate of the released Glc*N*Ac and site of its release (cytoplasm *versus* periplasm) are not yet known. In contrast to *M. smegmatis*, the other Mycobacteriales tested—*M*. *tuberculosis* and *M. abscessus*—cannot use Glc*N*Ac as a sole carbon source, cannot assimilate radiolabeled Glc*N*Ac, and/or do not encode homologs of the inner membrane Glc*N*Ac transporter/kinase NagE (27, 28, 92). Nonetheless, all three species label with 2-modified Mur*N*Ac probes (Figs. 2, 3 and S1) and the peptidoglycan of 2-alkNAM-treated *M. smegmatis* and *M. tuberculosis* contains the alkyne handle in Glc*N*Ac in addition to Mur*N*Ac. Thus, we speculate that a common pathway for funneling recycled Mur*N*Ac back into peptidoglycan involves uptake of Mur*N*Ac across the inner membrane by an unknown transporter followed by lactyl etherase generation of Glc*N*Ac and/or its intermediates in the cytoplasm.

In *E. coli*-type recycling, Mur*N*Ac is converted into both Glc*NA*c and glucosamine intermediates prior to peptidoglycan incorporation: MurP transports and phosphorylates Mur*N*Ac to Mur*N*Ac-6-P, MurQ removes the lactate to Glc*N*Ac-6-P, NagA deacetylates to Glc*N*-6-P, and GlmM and GlmU convert to UDP-Glc*N*Ac (Figs. 1 and 4, *E*, and 6) (16, 26, 33, 34, 35, 36, 37, 38, 39, 40, 41, 42). Recycled Glc*N*Ac is also converted into glucosamine intermediates: NagE transports and phosphorylates the sugar to Glc*N*Ac-6-P, followed by NagA, GlmM, and GlmU conversion to UDP-Glc*N*Ac (29, 30, 31, 32, 36). We find that native muramic acids rescue *glmS* or *glmM* depletion (Fig. 4) but that Glc*N*Ac rescues only *glmS* depletion. We speculate that in *M. smegmatis*, Glc*N*Ac is recycled as it is in *E. coli*, *i.e.* transported and phosphorylated by NagE to Glc*N*Ac-6-P. However, none of the Mycobacteriales tested here encode MurP homologs, and only *M. smegmatis* encodes MurQ. We therefore posit that a common pathway for at least a portion of recycled muramic acids to enter back into peptidoglycan synthesis includes transport and transformation of the sugar to a Glc*N*Ac intermediate other than Glc*N*Ac-6-P *via* an unidentified lactyl etherase, followed by additional, unidentified enzyme(s) that generate UDP-Glc*N*Ac (Fig. 6).

Muramic acid isolated from mycobacterial peptidoglycan can bear either *N*-acetyl (Mur*N*Ac) or *N*-glycolyl (Mur*N*Glyc; (28, 46, 47, 48)). Our data suggest that in *M. smegmatis* Mur*N*Ac and Mur*N*Glyc access the same pathways for incorporation: an *E. coli*-type pathway with glucosamine intermediates and a non-*E. coli*, non-*Pseudomonas* type pathway with Glc*N*Ac intermediates. Given that peptidoglycan glucosamine is known to be 2-modified with *N*-acetyl (Glc*N*Ac) but not *N*-glycolyl (Glc*N*Glyc), it is possible that *N*-glycolyl on recycled Mur*N*Glyc may be replaced by *N*-acetyl during the non-*E. coli*, non-*Pseudomonas* recycling process.

Cell envelope salvage and recycling can be vulnerable pathways for stressed, non/slow-replicating bacteria. In *M. tuberculosis*, for example, recycling of trehalose sugars from the outer mycomembrane occurs in *in vitro* models of stress, including hypoxia (12), biofilms (13, 14), and carbon deprivation (10, 11), and promotes survival in host macrophages (10), survival *in vivo* (15), and drug tolerance (93). Less is known about when and why mycobacteria recycle peptidoglycan sugars (26, 27, 28, 94). In other species, cell wall recycling mutants often do not have phenotypes under standard laboratory conditions (95, 96) but can have defects in stationary phase (16); sensitivity to certain cell wall-acting antibiotics (17, 18, 19, 20); impaired biofilm formation (21); attenuation *in vivo* (22, 23) and in macrophages (24); abnormal immune activation (25). Our data suggest that mycobacteria can harness a muramic acid recycling pathway that is distinct from previously-characterized *E. coli*- and *Pseudomonas*-type mechanisms. Future identification of the transporters and enzymes that mediate the Mycobacteria-type pathway prior to its intersection with *de novo* peptidoglycan biosynthesis will enable dissection of physiological significance during stress and evaluation as a potential therapeutic target.

## Experimental procedures

### Bacterial strains and culture conditions

*M. smegmatis* mc^2^155 (97) and *M. abscessus* ATCC 19977 were grown in Middlebrook 7H9 medium (Thermo Fisher Scientific) supplemented with bovine serum albumin (2.632% w/v), dextrose (1.053% w/v), sodium chloride (0.447% w/v), glycerol (0.4% v/v), and Tween-80 (0.05% v/v) at 37 °C and buffered or not with MOPS (Thermo Fisher Scientific, (98, 99)). See “*MurNAc, MurNGlyc, GlcNAc, and 2-alkNAM growth rescue experiments”* below for details. *C. glutamicum* ATCC 13032 was grown in brain heart infusion medium (Becton Dickinson). *M*. *tuberculosis* mc^2^6206 (H37Rv Δ*panCD* Δ*leuCD* (100)) was grown in 7H9 supplemented with 10% Middlebrook Oleic Albumin Dextrose Catalase, glycerol (0.5% v/v), and Tween-80 (0.05% v/v) at 37 °C. *E. coli lptD*4213 K-12 MG1655, a mutant with an impaired outer membrane (79, 101, 102, 103), and *E*. *coli* RFM795, a mutant unable to synthesize select outer membrane proteins, expressing *Tannerella forsythia* AmgK and MurU (55, 56, 101) were grown in lysogeny broth (Genesee Scientific) at 37 °C, while *A. globiformis* NRRL B-24224 SEA and *M. foliorum* NRRL B- 2979 SEA (University of Pittsburgh) were grown in lysogeny broth at 30 °C.

Libraries of ATC-inducible CRISPRi plasmids (built on the pJR962 backbone (85, 86)) and kanamycin- and zeocin-resistant *M*. *smegmatis* deletion mutants were provided by the *Mycobacterium* Systems Resource Database (MSRdb; (86)). pJR962 empty vector or pJR962 containing d*cas9* and single guide (sg) RNA sequences targeting *msmeg*_1568 (*glmS*), *msmeg*_1559 (*glmM*), or *msmeg*_5426 (*glmU*) were electroporated into *M. smegmatis* mc^2^155. The sgRNA of *glmU* (gatataatctgggaATGCGCGTGCCGGCTCCGGCGGgtttttgtactcga) was inserted into pJR962 using the NEBridge Golden Gate Assembly Kit (BsmBI-v2) per manufacturer recommendations with a 10:1 ratio of oligo to plasmid. Of note, *M*. *smegmatis* mc^2^155 has two copies of *murA* (*msmeg*_4932), as this gene is found in a duplicated region of the chromosome (104, 105). To enhance our ability to knock down *murA* expression, pJR962 bearing a *murA* sgRNA was electroporated into Δ*murA M*. *smegmatis*, an MSR deletion strain with one copy of *murA.* This generated Δ*murA +* pJR962-sg*murA*, in which one copy of *murA* is deleted and expression of the other can be depleted by CRISPRi.

A knockout of *msmeg*_0193, encoding an ortholog of the peptidoglycan recycling protein MurQ, was generated *via* the ORBIT strategy detailed by Murphy *et al.* (106). Briefly, WT *M*. *smegmatis* mc^2^155 was electroporated with the pKM461 targeting vector. Bacteria were incubated with 25 μg/ml kanamycin and 500 ng/ml ATC until reaching an OD_600_ of 1.0, after which electrocompetent cells were prepared by washing in increasingly smaller volumes of ice-cold 10% glycerol (Thermo Fisher Scientific). Freshly made competent *M. smegmatis* (200 μl) were electroporated with 2 μg of ORBIT oligo (Supporting Information) and 200 ng of pKM446 plasmid and transferred to 5 ml of 7H9, shaking at 37 °C overnight. The bacteria were centrifuged and plated onto 7H10 agar plates containing 50 μg/ml hygromycin, and successful gene knockout was verified *via* DreamTaq PCR using manufacturer’s recommendations (Thermo Fisher Scientific; Table 1). Colonies of Δ*murQ* were then electroporated with pKM512 for curing of antibiotic resistance. Bacteria were incubated at OD_600_ 0.1 with 500 ng/ml ATC until ∼OD_600_ 1.0, then sub-cultured to repeat this incubation. The bacteria were screened on 7H10 and 7H10 plus hygromycin (50 μg/ml) or zeocin (25 μg/ml) to detect knockout mutants sensitive to all antibiotics. pJR962-sg*glmS* was later electroporated into the cured Δ*murQ* strain.

**Table 1:** Primers used for identifying *msmeg*_0193 KO

Gene region	Primer (5′ - 3′)
Knocked-out Gene Region	
Forward	GACGACTCATCTACCTCGGC
Reverse	GACTCCACCACGATCCGC
5′ End of Gene/Insert	
Forward	CTGCCTTTTCGTGGGTGTTG
Reverse	TACCGCCTTTGAGTGAGCTG
3′ End of Gene/Insert	
Forward	TCACCTTCCTGCACGACTTC
Reverse	CCCCCTCTACGAGACGAAGA

### Metabolic labeling for microscopy and flow cytometry

Click reactions were modified from previous protocols (61, 63, 67, 81). Bacteria were grown until mid-log phase (OD_600_ ∼0.6–0.8) and treated for 30 min with either 2 mM alkyne-d-alanine-d-alanine (alkDADA, also known as EDADA, (61, 72), synthesized by WuXi AppTech), 2-alkyne- or 2-azide-Mur*N*Ac (2-alkNAM or 2-azNAM (50, 51)). Bacteria were centrifuged at 2147×*g* for 4 min at 4 °C and washed twice with PBS containing 0.05% Tween 80 (PBST; pre-chilled) using the same conditions. Bacteria were then fixed in 2% paraformaldehyde for 10 min, washed thrice with PBST containing 0.01% w/v bovine serum albumin (PBSTB), and resuspended for CuAAC. The CuAAC mix consisted of 1 mM copper sulfate, 128 μM Tris(benzyltriazolylmethyl)amine [TBTA], 1.2 mM sodium ascorbate (freshly prepared), and 20 μM fluorescent azide or alkyne (Click Chemistry Tools) in PBSTB diluent. Bacteria were shaken at 600 rpm for 30 min using a Benchmark Multi-Therm Heat Shake, then centrifuged, washed twice more with PBSTB and once with PBST, and finally resuspended in PBST prior to imaging on a Nikon Eclipse E600 microscope. Similar reactions were performed in which *M*. *smegmatis* was incubated with 2-tetrazine (Htz)-Mur*N*Ac probe and reacted with trans-cyclooctene fluorophore (Click Chemistry Tools) in PBSTB (55). Species other than *M. smegmatis* were grown to mid-log phase (OD_600_ ∼0.6–0.8) and incubated for ∼50% generation time ± 2 mM alkDADA or 6 mM 2-alkNAM. 2-alkNAM was prepared according to literature precedent and matched reported spectra (50, 53). All subsequent steps were repeated as described above.

Click reactions were performed in duplicate for three biological replicates of the following *M*. *smegmatis* strains: WT + pJR962-sg*glmM* or pJR962-sg*glmU* and Δ*murA* + pJR962-sg*murA*. Strains bearing pJR962-sg*glmM* were diluted to OD_600_ ∼0.01 while the remaining strains were diluted to ∼0.02 before either inducing depletion with 50 ng/ml ATC or treating with ethanol (EtOH) as the vehicle control. *M. smegmatis* with pJR962-sg*glmM*, pJR962-sg*glmU*, and pJR962-sg*murA* were incubated for 14 h, 11 h, or 9 h, respectively, to induce depletion and trigger a slight decrease in culture turbidity prior to incubation in probe as described above. Median fluorescence intensity was recorded using the 5-laser mode of a BD DUAL LSRFortessa, capturing 30,000 events. The change in fluorescence intensity was computed for each strain relative to the indicated negative controls.

### Phylogenetic tree

A phylogenetic tree was generated from 16S rRNA sequences obtained from NCBI (GenBank: *M. smegmatis*, AJ131761.1; *M. tuberculosis*, MH794239.1; *M. abscessus*, AB548599.1; *C. glutamicum*, AF314192.1; *A. glorbiformis*, AB089841.1; *M. foliorum*, AJ249780.1; *E. coli*, AB681728.1) using the List of Prokaryotic names with Standing in Nomenclature as guidance (107). A neighbor-joining tree was constructed with 1000 bootstrap replicates *via* MEGA12 using default settings.

### High-resolution mass spectrometry

A 10 ml culture of WT *M*. *smegmatis* was grown to OD_600_ ∼0.6, diluted four-fold, then split into 10 ml aliquots, each incubated shaking for 3 h at 37 °C ± 2 mM of alkyne-d-alanine (alkDA; R-propargylglycine; Thermo Fisher Scientific) or 5 mM 2-alkNAM. Bacteria were centrifuged at 3000 × *g* for 5 min, washed twice in 10 ml of PBS, then resuspended in 2.5 ml of digestion buffer (250 μl of 1 M Tris pH 7.9, 25 μl of 5 M NaCl, 20 μl of 0.5 M EDTA, and 4.75 ml of deionized water). Freshly prepared lysozyme (Sigma-Aldrich; 50 mg/ml, 20 μl) was added to each sample. Bacteria were shaken at 37 °C for 3 days with additional infusions of 20 μl of freshly prepared lysozyme every 12 h. To collect the peptidoglycan fragments released by lysozyme, bacteria were centrifuged at 3075 × *g* for 5 s and added to an Amicon Ultra 3K Filter. Samples were centrifuged at 3000 × *g* for 30 min to collect flowthrough. The filter was washed with 2 ml of deionized water and spun as described prior to collect the flowthrough. All flowthroughs were combined, flash frozen on dry ice, and lyophilized overnight. A similar protocol was performed for *M*. *tuberculosis*, except that bacteria were incubated with probes for 3 days (∼3.5 doublings). The lyophilized samples were dissolved in 50 μl of deionized water and loaded onto Acquity UPLC BEH C18 column 2.1 × 50 mm (Waters) using a S11 Dionex UHPLC. The samples were eluted with the following gradient: 0.5 ml/min – 1 linear gradient starting from 0% eluent A (0.1% formic acid in water) to 50% eluent B (formic acid in acetonitrile) in 6 min and subsequently underwent high-resolution mass analysis (when needed desired fragments were subjected to MS-2) on a Q-Exactive Orbitrap (Thermo Fisher Scientific). The Q-exactive Orbitrap MS parameters were set as follows: positive ionization mode, using the Full MS/DD-MS^2^ Top 10 experimental mode. Full scan range was 250 to 3000 m/z at a resolution of 35,000 and AGC target of 5^e6^ with max injection time of 200 ms. the top 10 most intense peaks were selected for fragmentation in a DD-MS^2^ experiment using stepped normalized collision energy of 12, 30 and 45 and isolation window of 2 m/z. The resolution for the MS^2^ scans was set to 17,500, AGC target of 1^e5^ and max injection time of 50 ms. All data were processed and analyzed on a Thermo Xcalibur Qual Browser. Exact masses and chemical formulas were calculated in Chemdraw Prime Version 26.0.0. *M. smegmatis* MS analysis was performed on one biological replicate and one technical replicate. *M. tuberculosis* MS sample analysis was performed on two biological replicates and one technical replicate of each.

### MurNAc, MurNGlyc, GlcNAc, and 2-alkNAM growth rescue experiments

Primary cultures of CRISPRi plasmid-bearing *M. smegmatis* were grown in 7H9 medium supplemented with 25 μg/ml kanamycin (and 25 μg/ml zeocin for Δ*murA +* pJR962-sg*murA*). Bacteria were back-diluted and grown overnight in fresh 7H9 medium buffered with 100 mM MOPS (Thermo Fisher Scientific, (98, 99)) without antibiotic to mid-log phase (OD_600_ ∼0.4–0.8). After adjusting the OD_600_ to 0.005 in MOPS-buffered 7H9, *M. smegmatis* were grown in duplicate or triplicate in 96-well flat-bottom plates ± 50 ng/ml ATC to induce expression of d*cas9* and sgRNAs. Plates were continuously shaken at 37 °C in a Biotek Synergy 2 plate reader at medium setting with hourly OD_600_ readings for 24 h. At 0 and 4 h, 5 mM Mur*N*Ac, Mur*N*Glyc, 2-alkNAM, Glc*N*Ac (Sigma-Aldrich), or vehicle control was added to the appropriate wells. Mur*N*Ac was prepared according to literature precedent and matched reported spectrum (108) or purchased from Sigma-Aldrich. Mur*N*Glyc was prepared according to literature precedent and described in Supporting Information (Fig. S13) (52, 109).

We initially observed that elevated concentrations of muramic acids (>5 mM) dampen growth of WT *M. smegmatis* and *M. tuberculosis* (Fig. S11, *A* and *B*) over the course of several generations. The sugars also acidified the 7H9 medium to varying degrees. Buffering the 7H9 with 100 mM MOPS (98, 99) rescued the growth defect, suggesting it was the result of low pH rather than the sugars. Thus, we performed long-term incubations in 5 mM Mur*N*Ac, Mur*N*Glyc, Glc*N*Ac, or 2-alkNAM (Figs. 4 and 5, *A*–*C*) in MOPS-buffered 7H9.

### Quantitative real-time PCR

Quantitative real-time PCR was performed similarly to Prithviraj *et al.* (110). WT *M. smegmatis* containing the pJR962 empty vector, pJR962-sg*glmM*, or pJR962-sg*glmU* were grown in 100 ml cultures ± 50 ng/ml of ATC until OD_600_ ∼0.4 to 0.6. Twenty-five OD units were pelleted for each treatment at 3200×*g* for 10 min. The supernatant was discarded, and the pellets were resuspended in 1 ml of TRIzol (Thermo Fisher Scientific) and transferred to tubes containing 0.5 ml of zirconia beads (Benchmark Scientific). Bacteria were bead-beat for 1 min with 2 min intervals on ice before incubating at room temperature for 5 min. The samples were spun for 30 s at 10,000 × *g*. The top TRIzol layer was transferred to a fresh RNAse-free tube, treated with 200 μl chloroform, shaken vigorously for 15 s, and left at room temperature for 10 min. The samples were spun at 12,000 × *g* for 10 min at room temperature and the top aqueous layer was saved. To each tube, 0.1 volumes of 3 M sodium acetate (pH 5.2, made with deionized, diethylpyrocarbonate-treated [DEPC] water) and 0.7 volumes of isopropanol were added. The tubes were inverted and stored at −20 °C for 2 h to precipitate nucleic acids. The samples were centrifuged at 16,000 × *g* for 30 min at 4 °C. The pellets were saved and washed twice in ice cold 70% ethanol (DEPC) using the same spin conditions. Samples were dried and the nucleic acids were resuspended in 40 μl DEPC-water (Sigma-Aldrich), 4 μl 10X TURBO DNase I buffer (Thermo Fisher Scientific), and 1 μl 1 U/μl DNase I (Thermo Fisher Scientific). The samples were vortexed and briefly spun at maximum speed before incubating for 2 h at 37 °C. RNA was further concentrated using Zymo RNA Clean and Concentrator following the manufacturer’s instructions (Zymo Research) and stored at −80 °C until cDNA synthesis. To generate cDNA, the different samples were processed using Invitrogen’s SuperScript IV Reverse Transcriptase according to manufacturer’s instructions, save that 50 μM random hexamers (New England Biotech), 1 μl of Promega RNasin Plus RNA inhibitor (Thermo Fisher Scientific), and 2 μM gene-specific reverse primers for *glmM*, *glmU*, and *gyrB* (*msmeg*_0005, housekeeping control (111)) were added (Table 2).

**Table 2:** Primers used for qRT-PCR reaction

Gene	Primer (5′ - 3′)
*gyrB*	
Forward	TATTCGGAGTCGGTGCACAC
Reverse	CTTGTCCTTGGCATACCGGT
*glmM*	
Forward	CCTGATGTCGCGCATGG
Reverse	CGCAACCGTGGTCTTGT
*glmU*	
Forward	TGGGACCGTTCACCTATCT
Reverse	ATCGTGCAGTTCTTGGTCTC

Each 20-μl reaction was composed of 10 μl ITaq Universal SYBR Green Supermix (Bio-Rad) with cDNA (final concentration 50 ng/μl) and appropriate primers (final concentration 200 nM; Table 2). Quantitative real-time PCR was performed using Opticon Monitor V 3.1 (Bio-Rad) on a DNA Engine Opticon 2 from MJ Research (Saint-Bruno-de-Montarville, Quebec). Reaction conditions were performed in technical triplicate as follows: 95 °C for 5 min; 40 cycles of incubations at 95 °C for 30 s, 67 °C for 30 s, taking reads after each cycle. A melting curve was performed from 50 °C to 95 °C. The 2^–ΔΔCT^ was calculated using C_T_ as the threshold cycle.

## Data availability

All data are contained within the article and Supporting Information.

## Supporting information

This article contains Supporting Information (50, 51, 52, 55, 108, 109).

## Conflict of interest

The authors declare that they have no conflicts of interest with the contents of this article.
