# Supplementary material for: Metabolic flexibility and an unusual route for peptidoglycan muramic acid recycling in mycobacteria
Source: J Biol Chem. 2026 Sep 18;302(11):113575. doi: 10.1016/j.jbc.2026.113575 (PMC13691567; doi:10.1016/j.jbc.2026.113575)
Supplement: Supplementary file 1 [file mmc1.docx]

**Metabolic flexibility and an unusual route for peptidoglycan muramic acid recycling in mycobacteria**

Stefanos Stravoravdis^1^, Bella R. Carnahan^2^, Rebecca A. Gordon^1,4^, Kimberly Wodzanowski^2^, Karina Havaleshko^1,3^, Elisabeth Fils-Aime^1^, Rachel Putnik^2^, Stephen Hyland^2^, Catherine Leimkuhler Grimes^2,5#^, M. Sloan Siegrist^1,4#^

^1^Department of Microbiology, University of Massachusetts, Amherst; Amherst, MA, USA.

^2^Department of Chemistry and Biochemistry, University of Delaware, Newark, Delaware 19716, United States.

^3^Department of Biochemistry and Molecular Biology, University of Massachusetts Amherst; Amherst, MA, USA.

^4^Molecular and Cellular Biology Graduate Program, University of Massachusetts, Amherst; Amherst, MA, USA.

^5^Department of Biological Sciences, University of Delaware, Newark, Delaware 19716, United States.

^#^Corresponding authors

**Supporting information**

In this document, we report information regarding oligo preparation for an ORBIT-mediated gene knockout, microscopy of additional Mur*N*Ac metabolic probes, LC-MS spectra, quantitative real-time PCR data, and additional growth curves of *Mycobacterium smegmatis* and *M. tuberculosis* supplemented with Mur*N*Ac and Glc*N*Ac.

**Supporting Experimental Information**

cccgcctgcgcgctcgatacgccgtgcggcctcgtcggcggagcagcccgccgcgagcatgacgatcgccgtGGTTTGTACCGTACACCACTGAGACCGCGGTGGTTGACCAGACAAACCgtcttcgtcgttcatgaccgccagcagctcggggacggtcagtcggtccagttccgtggtccgtgtgttgcg

Oligo used for ORBIT-mediated gene knockout of *msmeg*_0193 (*murQ*). The oligo has two flanking sequences of 72 bases with homology to *msmeg*_0193, leaving out an additional 30 bases of the gene flanking this homologous region. Due to the orientation and position of the gene in the genome, the reverse complement of the oligo was taken, yielding the above sequence, to target the chromosome lagging strand. The blue regions indicate homology to genomic DNA, whereas the purple is the *attP* site utilized for eventual replacement of the internal gene sequence with the pKM446 vector.

**Supporting Information: General Materials and Instrumentation**

*Materials*

All reagents were purchased from Sigma Aldrich, Fisher Scientific, ThermoFisher, Alfa

Aesar, or Oakwood Chemical and used without further purification, unless otherwise noted. All NMR solvents were purchased from Cambridge Isotope Laboratories, Inc.

Unless otherwise noted, all reactions were performed in flame or oven dried flasks equipped with rubber septa, positive pressure of nitrogen, and magnetic stirring. All solvents were anhydrous and transferred via stainless steel syringe or cannulae. Syntheses were monitored by electrospray ionization liquid chromatography mass spectrometry (ESI LC-MS) and thin layer chromatography (TLC) in which 250 µm glass plates coated with silica gel (Sorbtech Silica HD TLC plates w/ UV 254 or Supelco® TLC Silica Gel 60G F254 25 Glass Plates) were used and visualized with shortwave 254 nm UV light or developed upon heating with p-anisaldehyde. Flash chromatography was carried out on silica gel (60 Å, 40- 63 m, Sorbent Technologies), Silicycle Siliaflash P60 silica gel (40-63 μm, 60Å), or Biotage® Sfär C18 D- Duo Samplet® Support (100 Å, 30 µm,12 g) column.

*Instrumentation*

All NMR spectra were recorded on Bruker AV 400 MHz, Neo 400 MHz, AV III 600 MHz, and Neo 600 MHz spectrometers. Chemical shifts are reported in parts per million (ppm) and referenced to their residual non-deuterated solvents peaks: CDCl3 (1 H: 7.26 ppm, 13C: 77.16 ppm), Methanol-d4 (1 H: 3.31 ppm, 13C: 49.00 ppm), DMSO-d6 (1 H: 2.50 ppm, 13C:

39.52 ppm), and Deuterium Oxide (1 H: 4.79 ppm). Coupling constants (J) are reported to the nearest 0.1 MHz. Multiplicities are reported as follows singlet (s), doublet (d), triplet (t), quartet (q), multiplet (m), ‘broad’ (br), coupling constant in Hz, integration, and assignment for carbohydrates was based on two-dimensional COSY, HSQC and HMBC experiments. 13C NMR, for compounds, resonances are proton decoupled and an APT pulse sequence was used to determine the type of carbon as noted: methylene and quaternary (CH2 and C) carbons appear ‘up’ and methyl and methine (CH3 and CH) carbons appear ‘down’ 19F NMR data were collected with proton decoupling. High-resolution mass spectra (HR-MS), ESI mode, were obtained on a Thermo Q-Exactive Orbitrap or a Waters GCT Premier in the Mass Spectroscopy Facility at the Department of Chemistry and Biochemistry, University of Delaware.

**Synthesis of Mur*N*Glyc:**

**Mur*N*Glyc (Compound 3):** **1** and **2** were prepared according to literature precedent (52, 109). **1** (100.0 mg, 0.398 mmol, 1.0 eq) was suspended in MeOH (5.0 mL) along with K_2_CO_3_ (82.5 mg, 0.597 mmol, 1.5 eq) and stirred for 30 minutes at room temperature. Then **2** (102.8 mg, 0.478 mmol, 1.2 eq) was added and the reaction was stirred overnight at room temperature. Upon completion, the reaction was cooled to 0 ℃ and NaOMe (4.63 M in MeOH, 172 uL, 0.798 mmol, 2.0 eq) was added dropwise and stirred for another hour at 0 ℃. Then DCM was slowly added till the precipitant stopped forming, and the solid was filtered, washing with 5% MeOH/DCM. The crude solid was then condensed onto celite and purified by flash column chromatography (40% MeOH/EtOAc) to yield **3**. 68.8 mg, 0.226 mmol, 57% yield. ^1^H NMR (600 MHz, D_2_O) δ 5.47 (d, J = 2.6 Hz, 1H), 4.44 (q, J = 6.9 Hz, 1H), 4.15 (s, 2H), 3.86 – 3.84 (m, 1H), 3.81 (d, J = 5.2 Hz, 1H), 3.79 (t, J = 4.6 Hz, 1H), 3.77 (m, 2H), 3.61 (ddd, J = 10.1, 7.0, 1.5 Hz, 1H), 1.36 (d, J = 7.0 Hz, 3H). ^13^C NMR (151 MHz, D_2_O) δ 177.6, 97.7, 92.6, 82.8, 80.6, 79.0, 78.3, 74.4, 73.0, 71.7, 63.7, 63.5, 63.2, 62.9, 57.8, 56.2, 22.6, 21.3, 21.2. HRMS (ESI-Pos) for C_11_H_20_NO_9_^+^ [M+H]^+^ calculated: 310.11326, observed: 310.11262 (-2.05689 ppm).

**2-alkNAM (Compound 4): 4** was synthesized according to literature precedent and matched reported spectra (50, 53).

**Mur*N*Ac (Compound 6): 6** was purchased from Sigma-Aldrich or synthesized according to literature precedent and matched reported spectra (108).

**Supporting Figures**

**Figure S1. Mur*N*Ac probes label *Mycobacterium smegmatis* cell surface.** Bacteria were labeled with 2-alkNAM (A), 2-azNAM (B), or tetrazine (Htz)-Mur*N*Ac (C) for ~25% doubling time and subjected to copper-catalyzed alkyne-azide cycloaddition with corresponding azide-fluor (A) or alkyne-fluor (B) or to tetrazine ligation to trans-cyclooctene (TCO)-fluor (50-52, 55). Zoomed in images of labeled cells for Mur*N*Ac-treated bacteria are shown on the far right, wherein polar and septal labeling are denoted as “p” and “s”, respectively. Scale bars, 8 μm.

**Figure S2. HRMS-ESI+ spectra of disaccharide fragment from purified and digested sacculi from 2-alkNAM-treated *M. tuberculosis*.** Structure, expected mass, chemical formula, observed mass [m/z+1], and mass error (ppm).

**Figure S3. HRMS-ESI+ spectra of disaccharide fragment from purified and digested sacculi from 2-alkNAM-treated *M. tuberculosis*.** Structure, expected mass, chemical formula, observed mass [m/z+1], and mass error (ppm).

**Figure S4. HRMS-ESI+ spectra of disaccharide fragment from purified and digested sacculi from 2-alkNAM-treated *M. tuberculosis*.** Structure, expected mass, chemical formula, observed mass [m/z+1], and mass error (ppm).

**Figure S5. HRMS-ESI+ spectra of disaccharide fragment from purified and digested sacculi from Mur*N*Ac-treated *M. tuberculosis* (negative control).** Structure, expected mass, chemical formula, observed mass [m/z+1], and mass error (ppm). The fragment shown was not observed in the sample.

**Figure S6. HRMS-ESI+ spectra of disaccharide fragment from purified and digested sacculi from Mur*N*Ac-treated *M. tuberculosis* (negative control).** Structure, expected mass, chemical formula, observed mass [m/z+1], and mass error (ppm). The fragment shown was not observed in the sample.

**Figure S7. HRMS-ESI+ spectra of disaccharide fragment from purified and digested sacculi from Mur*N*Ac-treated *M. tuberculosis* (negative control).** Structure, expected mass, chemical formula, observed mass [m/z+1], and mass error (ppm). The fragment shown was not observed in the sample.

**Figure S8. HRMS-ESI+ spectra of disaccharide fragment from purified and digested from 2-alkNAM-treated *M. smegmatis* sacculi.** Structure, expected mass, chemical formula, observed mass [m/z+1], and mass error (ppm).

**Figure S9. HRMS-ESI+ spectra of disaccharide fragment from purified and digested from 2-alkNAM-treated *M. smegmatis* sacculi.** Structure, expected mass, chemical formula, observed mass [m/z+1], and mass error (ppm).

**Figure S10. HRMS-ESI+ spectra of disaccharide fragment from purified and digested from 2-alkNAM-treated *M. smegmatis* sacculi.** Structure, expected mass, chemical formula, observed mass [m/z+1], and mass error (ppm).

**Figure S11. Impact of Mur*N*Ac on mycobacterial growth in standard 7H9 medium.** (A) Wild-type *M. smegmatis* was treated with different concentrations of Mur*N*Ac (i) or Glc*N*Ac (ii) at 0 and 4 hours. (B) *M. tuberculosis* was treated with increasing concentrations of Mur*N*Ac (i) and Glc*N*Ac (ii) at days 0 and 2 during a week-long incubation. Experiments were performed in biological duplicate and technical triplicate in 7H9 medium without MOPS (compare to **Figure 4Ai-4Aii, 4Di-4Dii**).

**Figure S12. Gene expression in pJR962-sg*glmM* and pJR962-sg*glmU* +/- depletion.** RNA was extracted from wildtype + pJR962 empty vector, wildtype + pJR962-sg*glmM*, and wildtype + pJR962-sg*glmU M. smegmatis* treated or not with anhydrotetracycline (ATC). Quantitative real-time PCR (RT-qPCR) were performed on each sample in technical triplicate and biological duplicate, with two RT-qPCR reactions performed for each biological replicate. Fold change (2^-ΔΔCt^) was calculated using C_T_ as the threshold cycle, normalizing +ATC to -ATC treatments. Representative fold changes for one biological replicate for *glmM* expression (A) and *glmU* expression (B) are shown above.

**Figure S13.** ^1^H and ^13^C NMR of Mur*N*Glyc (Compound **3**)
